# Current Challenges and Future Directions in Recombinant AAV-Mediated Gene Therapy of Duchenne Muscular Dystrophy

**DOI:** 10.3390/ph6070813

**Published:** 2013-06-27

**Authors:** Takashi Okada, Shin'ichi Takeda

**Affiliations:** Department of Molecular Therapy, National Institute of Neuroscience, National Center of Neurology and Psychiatry, 4-1-1 Ogawa-Higashi, Kodaira, Tokyo 187-8502, Japan

**Keywords:** DMD, AAV, immune response

## Abstract

Various characteristics of adeno-associated virus (AAV)-based vectors with long-term safe expression have made it an exciting transduction tool for clinical gene therapy of Duchenne muscular dystrophy (DMD). Although host immune reactions against the vector as well as transgene products were detected in some instances of the clinical studies, there have been promising observations. Methods of producing AAV vectors for considerable *in vivo* experimentation and clinical investigations have been developed and a number of studies with AAV vector-mediated muscle transduction were attempted. Notably, an intravenous limb perfusion transduction technique enables extensive transgene expression in the skeletal muscles without noticeable adverse events. Furthermore, cardiac transduction by the rAAV9-microdystrophin would be promising to prevent development of cardiac dysfunction. Recent achievements in transduction technology suggest that long-term transgene expression with therapeutic benefits in DMD treatment would be achieved by the rAAV-mediated transduction strategy with an adequate regimen to regulate host immune response.

## 1. Introduction

Duchenne muscular dystrophy (DMD) is the most common form of childhood muscular dystrophy. DMD is an X-linked recessive disorder with an incidence of one in 3,500 live male births [1]. DMD causes progressive degeneration and regeneration of skeletal and cardiac muscles due to mutations in the *dystrophin* gene, which encodes a 427-kDa subsarcolemmal cytoskeletal protein [2]. DMD is associated with severe, progressive muscle weakness and typically leads to death between the ages of 20 and 35 years [3]. Due to recent advances in respiratory care, much attention is now focused on treating the cardiac conditions suffered by DMD patients.

**Figure 1:**
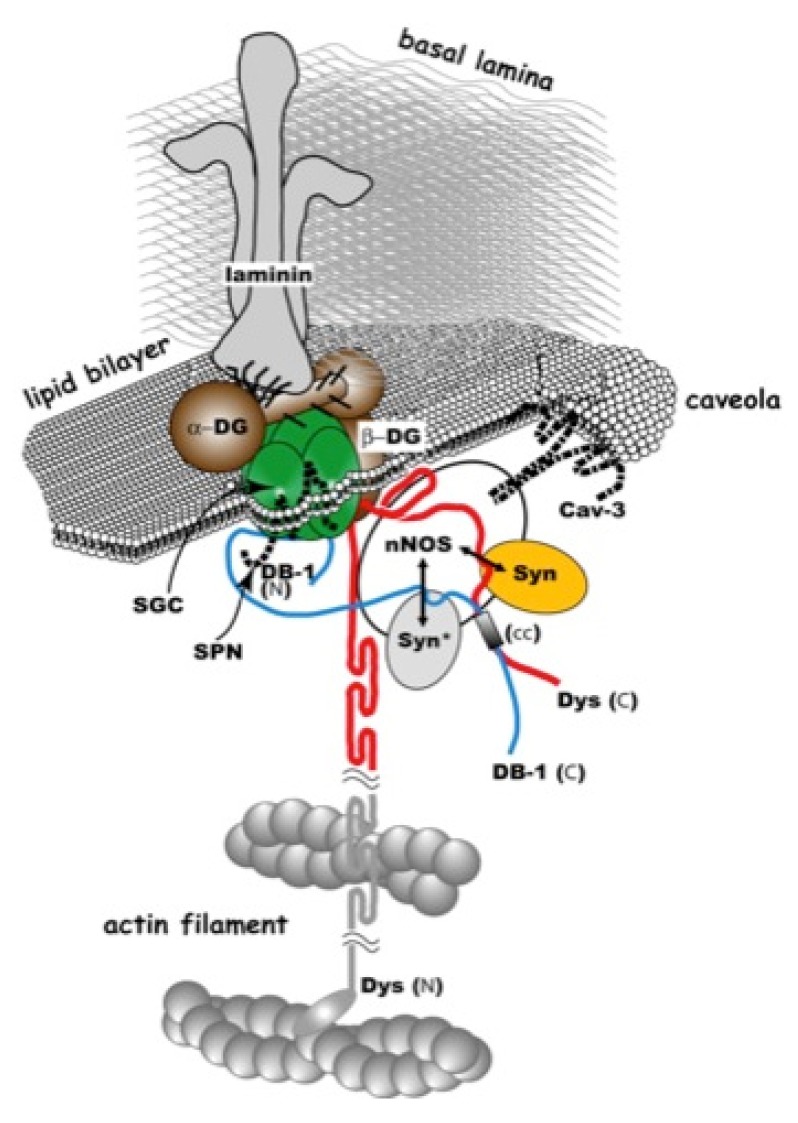
Dystrophin-glycoprotein complex. Molecular structure of the dystrophin-glycoprotein complex and related proteins superimposed on the sarcolemma and subsarcolemmal actin network (redrawn from Yoshida *et al*. [5], with modifications). cc, coiled-coil motif on dystrophin (Dys) and dystrobrevin (DB); SGC, sarcoglycan complex;SSPN, sarcospan; Syn, syntrophin; Cav3, caveolin-3; N and C, the N and C termini, respectively; G, G-domain of laminin; asterisk indicates the actin-binding site on the dystrophin rod domain; WW, WW domain.

The approximately 2.5-megabase *dystrophin* gene is the largest gene identified to date, and because of its size, it is susceptible to a high sporadic mutation rate. Absence of dystrophin and the dystrophin-glycoprotein complex (DGC) from the sarcolemma leads to severe muscle wasting (Figure 1). Whereas DMD is characterized by the absence of functional protein, Becker muscular dystrophy (BMD), which is commonly caused by in-frame deletions of the *dystrophin* gene, results in the synthesis of a partially functional protein. Therefore, BMD patients usually demonstrate a later onset and a slower progression of the muscular dystrophy, although severity of phenotypes is heterogeneous [4].

Various new strategies for DMD drug therapy are considered to use steroids, immunosuppressants, myostatin inhibitor, utrophin upregulator, and vitamin D [6]. The medication so far seems to be effective in delaying the disease progression is corticosteroid, which increases muscle strength in the randomised controlled studies. Deflazacort is an oxazoline derivative of prednisone and is as effective as prednisone in treating DMD. Randomised trial suggests reduced incidence and severity of obesity with deflazacort than with prednisone, although the risk of development of cataracts is elevated in the use of deflazacort. Since the benefit of corticosteroid treatment might be associated with the immunosuppressive effect, studies with other immunosuppressants involving cyclosporine-A were carried out. However, a placebo-controlled, double-blind study of 146 ambulant DMD patients who received cyclosporine-A or placebo alone and in combination with prednisone demonstrated no difference in muscle strength and functional abilities between the treatment groups [7].

Stop codon read-through drug could suppress termination codons by creating RNA misreading, allowing the insertion of alternative amino acids at the site of the mutated premature termination codon. Ataluren (PTC124) is an orally administered drug, which is bound to the 60s ribosomal subunit. Its efficacy in *mdx* mice is similar to gentamicin, producing dystrophin expression in 20-25% of muscle fibres [8]. With these results, a double-blind, randomised, multicentric study was carried out on 174 patients. After 48 weeks of taking low doses of ataluren, the patients showed some improvement in the 6 min walk test (http://clinicaltrials.gov).

By inducing the skipping of specific exons during mRNA splicing, antisense compounds correct the open reading frame of the DMD gene and thus to restore truncated yet functional dystrophin expression *in vitro* [9]. In fact, multi-exon skipping leading to an artificial DMD protein lacking the amino acids from exons 45 through 55 could rescue up to 63% of patients with DMD [10]. Intravenous infusion of an antisense phosphorothioate oligonucleotide created an in-frame *dystrophin* mRNA via exon skipping in a 10-year-old DMD patient possessing an out-of-frame exon 20 deletion of the *dystrophin* gene [11]. The adverse-event profile and local dystrophin-restoring effect of a single intramuscular injection of an antisense 2'-*O*-methyl phosphorothioate oligonucleotide, PRO051/GSK2402964, in patients with DMD were explored [12]. Four patients received a dose of 0.8 mg of PRO051 in the TA muscle. Each patient showed specific skipping of *dystrophin* gene exon 51 in 64% to 97% of myofibers, without clinically apparent adverse side effects. Weekly intravenous injections of considerably stable morpholino phosphorodiamidate (morpholino) antisense oligonucleotide induced functional levels of dystrophin expression in body-wide skeletal muscles of *mdx* mice, with concomitant improvement in muscle function [13]. Also, the efficacy and toxicity of intravenous morpholino-induced exon skipping were tested using CXMD_J_ dogs, and widespread rescue of dystrophin expression to therapeutic levels was observed [14]. Furthermore, a phosphorodiamidate morpholino oligomer with a designed cell-penetrating peptide can efficiently target a mutated *dystrophin* exon in cardiac muscles [15]. A chronic long-term administration of low-dose unmodified morpholino significantly ameliorates the muscular dystrophic phenotype and improves the activity of *mdx* mice [16]. A study targeting the exon 51 in seven DMD patients (AVI-4658) showed that those had received higher doses (0.9 mg) produced the dystrophin at 22%–32% levels of normal in 44%–79% of their muscle fibers [17]. In this study, no signs of toxicity were observed, while a previous study performed on non-human primates had shown tubular degeneration in the kidneys.

## 2. Gene-Replacement Strategies Using Virus Vectors

Although the mutation specific approach holds promise, development for patient subgroups is challenging as therapeutic effects on most mutations have not yet been elucidated. Therefore, gene replacement strategy to use universal therapeutic gene would be considered for monogenic diseases in which the gene product is either non-functional or is missing.

### 2.1. Choice of Vector

Although there are several considerations for any viral vector, successful DMD gene therapy requires an adequate level of long-term transgene expression in the muscle. Due to innovative cloning and preparation techniques, adenovirus vectors are efficient delivery systems of episomal DNA into eukaryotic cell nuclei [18]. The utility of adenovirus vectors has been increased by capsid modifications that alter tropism, and by the generation of hybrid vectors that promote chromosomal insertion [19]. Also, gutted adenovirus vectors devoid of all adenoviral genes allow for the insertion of large transgenes, and trigger fewer cytotoxic and immunogenic effects than do those only deleted in the E1 regions of the adenovirus early genes [20]. Human artificial chromosomes (HACs) have the capacity to deliver genes in any size into host cells without integrating the gene into the host genome, thereby preventing the possibility of insertional mutagenesis and genomic instability [21].

Long-term correction of genetic diseases requires permanent integration of therapeutic genes into chromosomes of the affected cells. However, retrovirus vector integration can trigger deregulated premalignant cell proliferation with unexpected frequency, most likely driven by retrovirus enhancer activity on the LMO2 gene promoter [22].

An adeno-associated virus (AAV)-based vector is emerging as the gene transfer vehicle with the most potential for use in the neuromuscular gene therapies. The advantages of the AAV vector include the lack of diseases associated with a wild-type virus, the ability to transduce non-dividing cells, and the long-term expression of the delivered transgenes [23]. Various serotypes of recombinant AAV (rAAV) exhibit a potent tropism for major organs including striated muscles (Table 1). Therefore, a supplementation of secretory protein can be achieved with this vector to use intramuscular injection [24]. Since a 5-kb genome is considered to be the upper limit for a single AAV virion, various truncated genes could be provided to meet size capacity, as may be necessary [25].

**Table 1:** Transduction efficiencies or representative rAAV serotypes in major tissues.

Tissue type	Effective serotype	Reference
Neurons and glial cells	AAV9, AAV7 > AAV8 > AAV5 > AAV2, AAV1	[26,27,28]
Glioblastoma	AAV8, AAV7 > AAV6 > AAV2 > AAV5	[28]
Cardiac tissue	AAV9 > AAV8 > AAV1, AAV6 > AAV2	[29,30,31,32]
Muscle (systemic)	AAV8	[33,34]
Muscle (local)	AAV1, AAV6	[33,35,36,37]
Liver (hepatocytes)	AAV9, AAV8	[38]
Pancreas	AAV8, AAV1	[39,40]
Retina	AAV8, AAV5 > AAV4 > AAV1, AAV2	[41,42,43]
Dendritic cells	AAV6	[44]
Hematopoietic stem cells	AAV1	[45]
Fibroblasts	AAV1, AAV6 > AAV2	[46]

The preparation of AAV vector for gene therapy study of neuromuscular diseases is greatly facilitated. Although AAV2 has been the serotype most extensively studied in preclinical and clinical trials, a number of primate AAV serotypes have been characterized in the literature and are designated. There is divergence in homology and tropism for various AAV serotypes. For instance, the homology with capsid protein is only about 60% between AAV2 and AAV5 [47], therefore the capsid structure could be responsible for the improved transduction efficiency. In fact, a significant difference in transduction efficiency of muscle by various AAV serotypes is recognized. We observed that intramuscular injection of AAV5-IL-10 promoted a much higher serum level of secreted transgene product, as compared to AAV2-mediated transfer [48]. We further demonstrated that AAV1 could more efficiently transduce the muscle than AAV5. Intramuscular single injection of modest doses of rAAV1 expressing IL-10 (6 × 10^10^ g.c. per rat) introduced therapeutic levels of the transgene expression over the long-term to treat pulmonary arterial hypertension [24]. Also, rAAV1-mediated sustained IL-10 expression significantly ameliorated hypertensive organ damage to improve survival rate of Dahl salt-sensitive rats [49]. Furthermore, this protein supplementation therapy by rAAV1-mediated muscle transduction was quite effective to prevent vascular remodeling and end-organ damage in the stroke-prone spontaneously hypertensive rat [50]. Interestingly, α-sarcoglycan expression with single intramuscular injection of rAAV8 was widely distributed in the hind limb muscle as well as cardiac muscle, and persisted for 7 months with a reversal of the muscle pathology and improvement in the contractile force in the alpha-sarcoglycan-deficient mice [34]. Intravenous administration of rAAV8 into the hind limb in dogs resulted in improved transgene expression in the skeletal muscles lasting over a period of eight weeks [51]. Moreover, rAAV9 would be administered systemically with excellent cardiac tropism [52]. Further strategies have been attempted to discover novel AAV capsid sequences from primate tissue, which can be used to develop newer-generation rAAVs with a greater diversity of tissue tropism for clinical gene therapy.

### 2.2. Modification of the Dystrophin GENE

The gutted adenovirus vector can package 14-kb of huge full-length *dystrophin* cDNA owing to the large deletion in the virus genome. Multiple proximal muscles of seven-day-old utrophin/dystrophin double knockout mice (*dko* mice), which typically show symptoms similar to human DMD, were effectively transduced with the gutted adenovirus bearing full-length murine *dystrophin* cDNA [53] However, further extraordinary improvements would be required to regulate the adenovirus-associated severe inflammation before clinical trials might be considered, since the adenovirus proteins elicit a more potent immune response *in vivo* compared to the AAV capsids [39].

**Figure 2:**
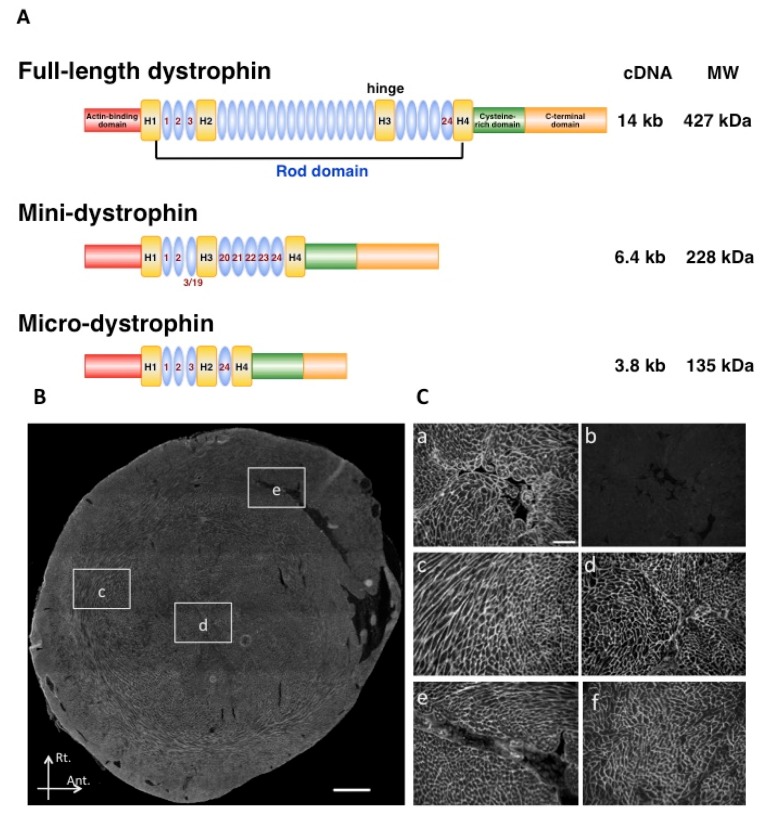
AAV9 vector-mediated *cardiac* transduction. (**A**) Structures of full-length and truncated dystrophin. Helper-dependent adenovirus vector can package 14-kb of full-length dystrophin cDNA because of the large-sized deletion in its genome. A mini-dystrophin is cloned from a patient with Becker muscular dystrophy, which is caused by in-frame deletions resulting in the synthesis of partially functional protein. A truncated micro-dystrophin cDNAs harboring only four rod repeats with hinge 1, 2, and 4 and a deleted C-terminal domain (delta CS1) is constructed to be packaged in the AAV vector. (**B**) Transverse section of *mdx* mouse heart at mid-ventricular level 24 weeks after rAAV9-mediated transduction of *micro-dystrophin*, stained with anti-dystrophin antibody NCL-DysB. Scale bar, 500 µm. (**C**) Expression of dystrophin in C57BL10 hearts at the sarcolemma (a), while it is absent in *mdx* hearts (b). Magnified views of sections from the center of the left ventricle at 28 weeks (c-e) show micro-dystrophin expression in the areas indicated in B (scale bar, 100 µm). At 74 weeks after transduction, *mdx* mice still retain extensive expression of micro-dystrophin (f).

A series of truncated *dystrophin* cDNAs containing rod repeats with hinge 1, 2, and 4 were constructed (Figure 2A) [25]. Although AAV vectors are too small to package the full-length *dystrophin* cDNA, AAV vector-mediated gene therapy using a rod-truncated *dystrophin* gene provides a promising approach [54]. The structure and, particularly, the length of the rod are crucial for the function of micro-dystrophin [55]. An AAV type 2 vector expressing micro-dystrophin (DeltaCS1) under the control of a muscle-specific MCK promoter was injected into the tibialis anterior (TA) muscles of dystrophin-deficient *mdx* mice [56], and resulted in extensive and long-term expression of micro-dystrophin that exhibited improved force generation. Likewise, AAV6 vector-mediated systemic *micro-dystrophin* gene transfer was effective in treating *dko* mice [57]. The potential for ameliorating the pathology of advanced-stage muscular dystrophy by systemic administration of AAV6 vectors encoding a micro-dystrophin expression construct was also demonstrated [58]. Furthermore, AAV9 vector-mediated systemic *micro-dystrophin* transduction of *mdx* mice accomplished prevention of cardiac fibrosis as well as heart failure [52]. The transduction efficiency achieved with rAAV9 was nearly complete, with persistent expression for 74 weeks after transduction (Figure 2B,C). Both the strong affinity of the rAAV9 for cardiac tissue and the therapeutic effect of the expressed micro-dystrophin might be involved in the prevention of the degeneration of the cardiomyocytes and cardiac fibrosis. However, when the aged mice (22-month-old) were treated, myocardial fibrosis was not mitigated despite the robust dystrophin expression by the rAAV9 [59].

The impact of codon usage optimization on micro-dystrophin expression and function in the *mdx* mouse was demonstrated to compare the function of two different configurations of codon-optimized *micro-dystrophin* genes under the control of a muscle-restrictive promoter (Spc5-12) [60]. In this study, codon optimization of micro-dystrophin significantly increased micro-dystrophin mRNA and protein levels after intramuscular and systemic administration of plasmid DNA or rAAV8 [60]. By randomly assembling myogenic regulatory elements into synthetic promoter recombinant libraries, several artificial promoters were isolated whose transcriptional potencies greatly exceed those of natural myogenic and viral gene promoters [61].

### 2.3. Use of Surrogate Genes

An approach using a surrogate gene would bypass the potential immune responses associated with the delivery of exogenous dystrophin. Methods to increase expression of utrophin, a dystrophin paralog, show promise as a treatment for DMD. A pharmacological agent for utrophin transcriptional upregulation demonstrated significant disease modifying effects in DMD mice [62]. The Phase 1 dose-escalating trial was conducted in healthy volunteers and evaluated a new aqueous formulation of SMT C1100, showing the formulation to be safe and well tolerated at all doses. Recombinant AAV (rAAV2/6) harboring a murine codon-optimized micro-utrophin transgene was intravenously administered into adult *dko* mice to alleviate the pathophysiological abnormalities [63]. The paralogous gene efficiently acted as a surrogate for *dystrophin*. However, full-length utrophin cannot anchor nNOS to the sarcolemma and utrophin gene overexpression failed to protect *mdx* muscle from exercise-associated injury [64]. To enhance efficacy of utrohin-based therapies, further innovative strategies would be required to avoid functional ischemia in association with the absence of sarcolemmal nNOS. Interestingly, although nNOS expression is decreased in the *dko* mice hearts, L-arginine transporter expression and function are significantly increased, suggesting a compensatory mechanism of the NO pathway and a potential entry site for therapeutics [65]. Myostatin is extensively documented as being a negative regulator of muscle growth. Systemic gene delivery of myostatin propeptide, a natural inhibitor of myostatin, enhanced body-wide skeletal muscle growth in both normal and *mdx* mice [66]. The delivery of various growth factors, such as insulin-like growth factor-I (IGF-I), has been successful in promoting skeletal muscle regeneration after injury [67]. 

In the formation, remodeling and degradation of extracellular matrix (ECM) components in pathological processes, matrix metalloproteinases (MMPs) are key regulatory molecules. MMP-9 is involved predominantly in the inflammatory process during muscle degeneration [68]. In contrast, MMP-2 is associated with ECM remodeling during muscle regeneration and fiber growth. Interestingly, modified tendon fibroblasts expressing an angiogenic factor (placenta growth factor) and an MMP-9 restored a vascular network and reduced collagen deposition, allowing efficient cell therapy in aged dystrophic mice [69].

## 3. AAV-Mediated Transduction of Large Animal Models

To gain acceptance as a preclinical study using large animal models or a medical treatment with a dose of over 1 × 10^13^ genome copies (g.c.)/kg body weight, transduction strategies with AAV vectors require a scalable and provident production. However, the production and purification of recombinant virus stocks with conventional techniques entails cumbersome procedures not suited to the clinical setting. Therefore, development of effective large-scale culture and purification steps are required to meet end-product specifications.

### 3.1. Vector Production

A production protocol of AAV vectors in the absence of a helper virus [70] is widely employed for triple plasmid transduction of human embryonic kidney 293 cells [23]. The adenovirus regions that mediate AAV vector replication (namely, the VA, E2A and E4 regions) were assembled into a helper plasmid. When this helper plasmid is co-transfected into 293 cells along with plasmids encoding the AAV vector genome and rep-cap genes, the AAV vector is produced as efficiently as when using adenovirus infection. Importantly, contamination of most adenovirus proteins can be avoided in AAV vector stock made by this helper virus-free method. Although various subtypes of the 293 cells harbor the E1 region of the adenovirus type 5 genome, to utilize a 293 cell stably expressing Bcl-xL (293-B) has great advantage to support E1B19K function and protect cells from apoptosis [71]. Despite improvements in vector production, including the development of packaging cell lines expressing Rep/Cap or methods to regulate Rep/Cap [72], maintaining such cell lines remains difficult, as the early expression of Rep proteins is toxic to cells.

We developed a large-scale transfection method of producing AAV vectors with an active gassing system that uses large culture vessels and 293-B cells to process labor-effective transfection in a closed system [73]. This vector production system achieved reasonable production efficiency by improving gas exchange to prevent pH drop in the culture medium. Also, vector purification with the dual ion-exchange membrane adsorbers was effective and allowed higher levels of gene transfer *in vivo* [74]. Furthermore, the membrane adsorbers enabled the effective recovery of the AAV vector in the supernatant exosomes of the transduced cells culture. The final titer of the purified vectors from a 10-tray flask with an active gassing apparatus usually ranges around 1 × 10^14^ g.c., although it depends on the vector constructs and transgene. This rapid and scalable viral purification protocol is particularly promising for considerable *in vivo* experimentation and clinical investigations.

### 3.2. Canine Models for the Gene Transduction Study

Dystrophin-deficient canine X-linked muscular dystrophy was found in a golden retriever with a 3’ splice-site point mutation in intron 6 [75]. The clinical and pathological characteristics of dystrophic dogs are more similar to those of DMD patients than are those of *mdx* mice. A beagle-based model of canine X-linked muscular dystrophy, which is smaller and easier to handle than the golden retriever-based muscular dystrophy dog (GRMD) model, has been established in Japan, and is referred to as canine X-linked muscular dystrophy in Japan (CXMD_J_) [76]. The limb and temporal muscles of CXMD_J_ dogs are affected by the time the dogs are two-months-old, which is the age corresponding to the second peak of serum creatine kinase.

Interestingly, we found extensive lymphocyte-mediated immune responses to rAAV2-*lacZ* after direct intramuscular injection into CXMD_J_ dogs, despite successful delivery of the same viral construct into mouse skeletal muscle [77]. In contrast to rAAV2-*lacZ*, rAAV8-*lacZ* transfer to canine skeletal muscles produced significantly higher transgene expression with less lymphocyte proliferation [51]].

It is increasingly important to develop strategies to treat DMD that consider the effect on cardiac muscle. The pathology of the conduction system in CXMD_J_ was analyzed to establish the therapeutic target for DMD [78]. Although dystrophic changes of the ventricular myocardium were not evident at the age of 1 to 13 months, Purkinje fibers showed remarkable vacuolar degeneration when dogs were as young as four-months-old. Furthermore, degeneration of Purkinje fibers was coincident with overexpression of Dp71 at the sarcolemma. The degeneration of Purkinje fibers could be associated with the distinct deep Q waves present in ECGs and the fatal arrhythmias seen in cases of dystrophin deficiency.

### 3.3. Immunological Issues of rAAV

**Figure 3:**
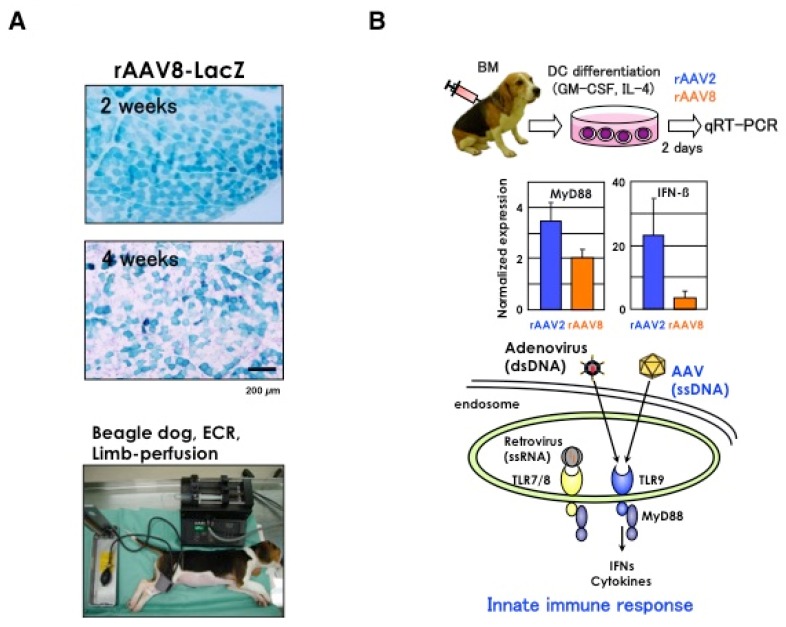
rAAV-mediated transduction of dog. (**A**) Intravascular vector administration by limb perfusion. A blood pressure cuff is applied just above the knee of an anesthetized CXMD_J_ dog. A 24-gauge intravenous catheter is inserted into the lateral saphenous vein, connected to a three-way stopcock, and flushed with saline. With a blood pressure cuff inflated to over 300 mmHg, saline (2.6 mL/kg) containing papaverine (0.44 mg/kg, Sigma-Aldrich, St. Louis, MO) and heparin (16 U/kg) is injected by hand over a 10 second period. The three-way stopcock is connected to a syringe containing rAAV8 (1 × 10^14^ vg/kg, 3.8 mL/kg). The syringe is placed in a PHD 2000 syringe pump (Harvard Apparatus, Edenbridge, UK). Five minutes after the papaverine/heparin injection, rAAV8-LacZ is injected at a rate of 0.6 mL/sec. Two minutes after the rAAV injection, the blood pressure cuff is released and the catheter is removed. Four weeks after the transduction, the expression slightly fell off. (**B**) AAV-mediated stimulation of innate immune response via TLR9/MyD88 pathway. Bone marrow (BM)-derived dendritic cells (DCs) were obtained from humerus bones and cultured in RPMI (10% FCS, p/s) for 7 days with canine GM-CSF and IL-4. DCs were transduced with rAAV2- or rAAV8-*lacZ* (1 × 10^6^ vg/cell for 4 h, and mRNA levels of MyD88 and IFN-β were analyzed. Untransduced cells were used as a normalization standard to demonstrate relative value of expression. Results are representative of two independent experiments. Error bars represent s.e.m., n = 3.

One of the biggest challenges facing AAV gene delivery is the host immune response. Neo-antigens introduced by AAV vectors evoke significant immune reactions in DMD muscle, since increased permeability of the DMD muscle allows leakage of the transgene products from the dystrophin-deficient sarcolemma of muscle fibers [79]. rAAV2 transfer into skeletal muscles of normal dogs resulted in low levels of transient expression, together with intense cellular infiltration, and the marked activation of cellular and humoral immune responses [77]. Furthermore, an *in vitro* interferon-gamma release assay showed that canine splenocytes respond to immunogens or mitogens more strongly than do murine splenocytes. Therefore, co-administration of immunosuppressants, cyclosporine (CSP) and mycophenolate mofetil (MMF) was attempted to improve rAAV2-mediated transduction. The AAV2 capsids can induce a cellular immune response via MHC class I antigen presentation with a cross-presentation pathway [80], and rAAV2 could also stimulate human dendritic cells (DCs) [81]. Whereas the non-immunogenic nature of AAV6 in murine studies, rAAV6 also elicited robust cellular immune responses in dogs [82]. In contrast, other serotypes, such as rAAV8, induce T-cell activation to a lesser degree [51]. The rAAV8-injected muscles showed lowed rates of infiltration of CD4^+^ and CD8^+^ T lymphocytes in the endomysium than the rAAV2-injected muscles [51]. Resident antigen-presenting cells, such as DCs, myoblasts, myotubes and regenerating immature myofibers, should play a substantial role in the immune response against rAAV. Our study also showed that MyD88 and co-stimulating factors, such as CD80, CD86 and type I interferon, are up-regulated in both rAAV2- and rAAV8-transduced dog DCs (Figure 3A) [51]. 

While low immunogenicity was considered a major strength supporting the use of rAAV in clinical trials, a number of observations have recently provided a more balanced view of this procedure [83]. An obvious barrier to AAV transduction is the presence of circulating neutralizing antibodies that prevent the virion from binding to its cellular receptor [84]. This potential threat can be reduced by prescreening patients for AAV serotype-specific neutralizing antibodies or by performing therapeutic procedures such as plasmapheresis before gene transfer. Another challenge recently revealed is the development of a cell-mediated cytotoxic T-cell (CTL) response to AAV capsid peptides. In the human factor IX gene therapy trial in which rAAV was delivered to the liver, only short-term transgene expression was achieved and levels of therapeutic protein declined to baseline levels 10 weeks after vector infusion [83]. This was accompanied by elevation of serum transaminase levels and a CTL response toward specific AAV capsid peptides. To overcome this response, transient immunosuppression may be required until AAV capsids are completely cleared. Additional findings suggest that T-cell activation requires AAV2 capsid binding to the heparan sulfate proteoglycan (HSPG) receptor, which would permit virion shuttling into a DC pathway, as cross-presentation [85]. Exposure to vectors from other AAV clades, such as AAV8, did not activate capsid-specific T-cells.

To establish the feasibility of multiple AAV1 injections for extending the treatment to whole body muscles, the dystrophic *mdx* mouse was repeatedly transduced with AAV1 vector, and the immune response was characterized [86]. By blocking the T-B crosstalk with anti-CD40 Abs and CTLA4/Fc fusion protein, a five-day-long immunomodulation treatment was found to be sufficient for totally abrogating the formation of anti-AAV1 antibodies. In a clinical trial for AAV-1-mediated gene transfer to muscle for lipoprotein lipase deficiency, T-cell responses directed to the AAV-1 capsid are dose-dependent [35]. However, whether they also limit the duration of expression of the transgene at higher doses is unclear. Also, a phase 1, open-label, dose-escalation clinical trial using an AAV-1 vector expressing normal AAT by intramuscular injection was performed [36]. Their findings suggest that immune responses to AAV capsid that develop after intramuscular injection do not completely eliminate transduced cells, despite development of neutralizing antibody and IFN-gamma enzyme-linked immunospot responses to AAV1 capsid at day 14 in all subjects. Actually, the heparin-binding ability of AAV2 and AAV6 does not determine the induction of T-cell responses following intramuscular injection in dogs [37].

To regulate host immune response against vectors and transgene products, treatments involving immunosuppressants and other strategies have been attempted in the animal models. A brief course of immunosuppression with a combination of anti-thymocyte globulin (ATG), CSP and MMF was effective in permitting AAV6-mediated, long-term and robust expression of a canine micro-dystrophin in the skeletal muscle of a dog DMD model [87]. Furthermore, this intramuscular injection of AAV6-canine micro-dystrophin in dystrophic dogs with a brief course of immunosuppressants demonstrated a robust dystrophin expression for at least two years and it was associated with molecular reconstitution of the dystrophin-glycoprotein complex at the muscle membrane [88]. Alternatively, the use of plasmapheresis and its possible association with pharmacological immunosuppressive treatments may help to design optimal management of seropositive patients for AAV gene therapy treatments [89]. 

### 3.4. Intravascular Vector Administration by Limb Perfusion

Although recent studies suggest that vectors based on AAV are capable of body-wide transduction in rodents [34], translating the characteristics into large animals with advanced immune system remains a lot of challenges. Intravascular delivery can be performed as a form of limb perfusion, which might bypass the immune activation of DCs in the injected muscle [90]. We performed limb perfusion-assisted intravenous administration of rAAV8-lacZ into the hind limb of Beagle dogs (Figure 3B) [51]. Administration of rAAV8 by limb perfusion demonstrated extensive transgene expression in the distal limb muscles of canine X-linked muscular dystrophy in Japan (CXMD_J_) dogs without obvious immune responses for the duration of the experiment over four weeks after injection. Also, the isolated limb perfusion administration of AAV8 vectors encoding human acid-alpha-glucosidase (GAA) effectively transduced the hindlimb muscles in GAA-knockout Pompe disease mice with a reduction of glycogen storage [91]. Furthermore, a protocol in rhesus macaque for the isolated focal limb perfusion targeting the vascular bed of the gastrocnemius was reported [92]. The vascular anatomy of nonhuman primates more clearly parallels humans to provide an appropriate substrate for translational experiments. Thus, the isolated limb perfusion administration would enhance AAV transduction of multiple skeletal muscles while reducing the required dosages in terms of vector particle numbers.

### 3.5. Global Muscle Therapies

In comparison with fully dystrophin-deficient animals, targeted transgenic repair of skeletal muscle, but not cardiac muscle, paradoxically elicits a five-fold increase in cardiac injury and dilated cardiomyopathy [93]. Because the dystrophin-deficient heart is highly sensitive to increased stress, increased activity by the repaired skeletal muscle provides the stimulus for heightened cardiac injury and heart remodeling. In contrast, a single intravenous injection of AAV9 vector expressing micro-dystrophin efficiently transduces the entire heart in neonatal *mdx* mice, thereby ameliorating cardiomyopathy [94].

Since a number of muscular dystrophy patients can be identified through newborn screening in future, neonatal transduction may lead to an effective early intervention in DMD patients. After a single intravenous injection, robust skeletal muscle transduction with AAV9 vector throughout the body was observed in neonatal dogs [95]. Systemic transduction was achieved in the absence of pharmacological intervention or immune suppression and lasted for at least six months, whereas rAAV9 was barely transduced into the cardiac muscle of dogs. Likewise, *in utero* gene delivery of full-length murine *dystrophin* to *mdx* mice using a high-capacity adenoviral vector resulted in effective protection from cycles of degeneration and regeneration [96]. 

## 4. Safety and Potential Impact of Clinical Trials

### 4.1. Clinical Trials for Muscle Transduction

The initial clinical studies lay the foundation for future studies, providing important information about vector dose, viral serotype selection, and immunogenicity in humans. The first virus-mediated gene transfer for muscle disease was carried out for limb-girdle muscular dystrophy type 2D using rAAV1. The study, consisting of intramuscular injection of virus into a single muscle, was limited in scope and the main conclusion was to establish the safety of this procedure in phase I clinical trials. The first clinical gene therapy trial for DMD began in March 2006 [97]. This was a Phase I/IIa study in which an AAV vector was used to deliver micro-dystrophin to the biceps of boys with DMD. The study was conducted on six boys with DMD, each of whom received an injection of mini-dystrophin-expressing rAAV2.5 in a muscle of one arm and a placebo in the other arm. Dystrophin-specific T cells were detected after treatment, providing evidence of transgene expression even when the functional protein was not visualized in skeletal muscle [98]. Circulating dystrophin-specific T cells were unexpectedly detected in two patients before vector treatment, since revertant dystrophin fibers expressing truncated dystrophin contained epitopes targeted by the autoreactive T cells [98]. The potential for T-cell immunity to self and non-self dystrophin epitopes should be considered in designing and monitoring experimental therapies for this disease. Basically, this issue is in common with the treatment of genetic diseases. A single dose of a self-complementary AAV8 vector expressing a codon-optimized human factor IX (FIX) transgene (scAAV2/8-LP1-hFIXco) was infused into a peripheral vein in six patients with severe hemophilia B [99]. Peripheral-vein infusion of the vector resulted in FIX transgene expression at levels sufficient to improve the bleeding phenotype, with few side effects. Although immune-mediated clearance of AAV-transduced hepatocytes remains a concern, this process may be controlled with a short course of glucocorticoids without loss of transgene expression. Although concerns regarding risk of an immune response to the transgene product limited the ability to achieve therapeutic efficacy, rAAV2-mediated gene transfer to human skeletal muscle can persist for up to a decade [100].

### 4.2. Gene Therapy Medicine

After more than two decades of expectations, the field of gene therapy appears close to reaching a regulatory approval by proposing rAAV-mediated muscle transduction. European medicine agency eventually recommends first gene therapy medicine for approval (http://www.ema.europa.eu/ema). The European Medicines Agency’s Committee for Medicinal Products for Human Use has recommended the authorization of Glybera (rAAV1-expressing LPL S447X variant) for marketing in the European Union. It is intended to treat lipoprotein lipase deficiency in patients with severe or multiple pancreatitis attacks, despite dietary fat restrictions. 

## 5. Future Perspectives

### 5.1. Modification of mRNA Splicing with rAAV-mediated Exon-Skipping

Long-term benefits would be obtained through the use of AAV vectors expressing antisense sequences to recover dystrophin expression through exon-skipping. The sustained production of dystrophin at physiological levels in entire groups of muscles as well as the correction of muscular dystrophy were achieved by treatment with an intramuscular or intra-arterial administration of AAV1-U7 to induce exon-skipping in mdx mice [101]. Also, persistent exon skipping, dystrophin rescue and functional benefit were observed as long as 74 weeks after a single systemic transduction of AAV-mediated antisense-U1 small nuclear RNA [102].

### 5.2. Pharmacological Intervention

The use of a histone deacetylase (HDAC) inhibitor depsipeptide effectively enhances the utility of rAAV-mediated gene therapy [103]. In contrast to adenovirus-mediated transduction, the improved transduction with rAAV induced by the depsipeptide is due to enhanced transgene expression rather than to increased viral entry. The enhanced transduction is related to the histone-associated chromatin form of the rAAV concatemer in the transduced cells. Since various HDAC inhibitors are approved in clinical usage for many diseases to achieve therapeutic benefits, the application of such inhibitors to the rAAV-mediated gene therapy is theoretically and practically reasonable.

### 5.3. Capsid Modification

A DNA shuffling-based approach for developing cell type-specific vectors is an intriguing possibility to achieve altered tropism. Capsid genomes of AAV serotypes 1–9 were randomly reassembled using PCR to generate a chimeric capsid library [104]. A single infectious clone (chimeric-1829) containing genome fragments from AAV1, 2, 8, and 9 was isolated from an integrin minus hamster melanoma cell line previously shown to have low permissiveness to AAV. Molecular modeling studies suggest that AAV2 contributes to surface loops at the icosahedral threefold axis of symmetry, while AAV1 and 9 contribute to two-fold and five-fold symmetry interactions, respectively.

A versatile rAAV targeting system to redirect rAAV-mediated transduction to specific cell surface receptors would be useful. Insertion of an IgG binding domain of protein A into the AAV2 capsid at amino acid position 587 could permit antibody-mediated vector retargeting, although producing mosaic particles is required to avoid low particle yields [105]. Alternatively, a targeting system using the genetic fusion of short biotin acceptor peptide along with the metabolic biotinylation via a biotin ligase was developed for the purification and targeting of multiple AAV serotypes [106].

### 5.4. AAV-Mediated Gene and Cell Therapy

There have been numerous reports to develop the therapeutic potential of Mesenchymal stem cells (or mesenchymal multipotent stromal cells, MSCs) [107]. Because of their immunomodulatory properties, increasing experimental and early clinical observations indicate that allogeneic, and even xenogeneic, MSCs may be useful for tissue transplantation [108]. Despite their heterogeneity and lack of defining markers, the MSCs have attracted so much translational attention as increasing evidence points to their predominant effect being not by donor differentiation but via paracrine mediators and exosomes [109]. Also, the immune tolerance with MSCs is well investigated in various animal studies. Infusion of syngeneic MSCs into a sensitized mouse model of kidney transplantation resulted in the expansion of donor-specific T-regulatory cells into lymphoid organs, prolonged allograft survival and promoted the development of tolerance [110].

Transplantation of genetically modified rAAV-producing cells is a possible future treatment for monogenic diseases as an *in situ* gene therapy. MSCs are known to accumulate at the site of inflammation or tumors, and therefore can be utilized as a platform for the targeted delivery of therapeutic agents [111]. The MSCs-based targeted gene therapy should enhance the therapeutic efficacy, since MSCs would deliver therapeutic molecules in a concentrated fashion. This targeted therapy can also reduce systemic adverse side effects, because the reagents act locally without elevating their systemic concentrations. We developed the genetically-modified MSCs that produce viral vectors to augment therapeutic efficacy of systemic gene therapy [112]. MSCs isolated from the SD rat bone marrow were transfected with retroviral vector components by nucleofection. As a result, the injection of luciferase-expressing vector-producing MSCs caused significantly stronger signal of bioluminescence at the site of subcutaneous tumors in mice compared with luciferease-expressing non-vector-producing MSCs [113]. Furthermore, tumor-bearing nude mice were treated with the vector-producing MSCs combined with HSV-*tk*/GCV system to demonstrate improved anti-tumor effects. This study suggests the effectiveness of vector-producing MSCs in systemic gene therapy. The therapeutic benefit of this strategy should be further examined by using rAAV-producing MSCs in the various animal models of inflammatory diseases including neuromuscular disorders.

Mesoangioblasts, vessel-associated stem cells, are promising candidates for future stem cell therapy of DMD. Intra-arterial delivery of wild-type canine mesoangioblasts results in an extensive recovery of dystrophin expression, normal muscle morphology and function [114]. Also, the aorta-derived mesoangioblasts delay or prevent development of dilated cardiomyopathy in dystrophin-deficient heart of *mdx* mice, although timing of transplantation is critical for achieving benefit with cell therapy in DMD cardiac muscle [115].

### 5.5. Targeted Vector Integration

A goal in clinical gene therapy is to develop gene transfer system that can integrate exogenous therapeutic genes at specific chromosomal loci as a safe harbor, so that insertional oncogenesis is prevented. AAV can insert its genome into a specific locus, designated AAVS1, on chromosome 19 of the human genome [116]. The AAV Rep78/68 proteins and the Rep78/68-binding sequences are the trans- and cis-acting elements needed for this reaction. A dual high-capacity adenovirus-AAV hybrid vector with full-length human dystrophin-coding sequences flanked by AAV integration-enhancing elements was tested for targeted integration [117]. Introduction into human cells of chimeric genomes, which contain a structure reminiscent of AAV proviral DNA, resulted in AAV Rep-dependent targeted DNA integration into the AAVS1 locus on chromosome 19.

## 6. Conclusions

To translate AAV-mediated transduction technologies into clinical practice in DMD therapy, development of an effective delivery system with improved vector constructs as well as efficient immunological modulation must be established. Besides, although an increasing number of scalable methods for purification of rAAV have been described, we need to further improve a large-scale GMP-compatible system for production and purification. A novel protocol that considers all of these issues would help improve the therapeutic benefits of DMD gene therapy.
